# The Characterization of Obese Polycystic Ovary Syndrome Rat Model Suitable for Exercise Intervention

**DOI:** 10.1371/journal.pone.0099155

**Published:** 2014-06-06

**Authors:** Chuyan Wu, Feng Lin, Shuwei Qiu, Zhongli Jiang

**Affiliations:** Department of Rehabilitation Medicine, The First Affiliated Hospital of Nanjing Medical University, Nanjing, China; University of Warwick – Medical School, United Kingdom

## Abstract

**Objective:**

To develop a new polycystic ovary syndrome (PCOS) rat model suitable for exercise intervention.

**Method:**

Thirty six rats were randomly divided into three experimental groups: PCOS rats with high-fat diet (PF, n = 24), PCOS rats with ordinary diet (PO, n = 6), and control rats with ordinary diet (CO, n = 6). Two kinds of PCOS rat model were made by adjustment diet structure and testosterone injection for 28 days. After a successful animal model, PF model rats were randomly assigned to three groups: exercise with a continuation of high-fat diet (PF-EF, n = 6), sedentary with a continuation of high-fat diet (PF-SF, n = 6), exercise with an ordinary diet (PF-EO, n = 6). Fasting blood glucose (FBG) and insulin (FINS), estrogen (E2), progesterone (P), and testosterone (T) in serum were determined by RIA, and ovarian morphology was evaluated by Image-Pro plus 6.0.

**Results:**

Body weight, Lee index, FINS increased significantly in PF rat model. Serum levels of E_2_ and T were significantly higher in PF and PO than in CO. Ovary organ index and ovarian areas were significant lower in PF than in CO. After intervention for 2 weeks, the levels of 1 h postprandial blood glucose (PBG_1_), 2 h postprandial blood glucose (PBG_2_), FINS and the serum levels of T decreased significantly in PF-EF rats and PF-EO rats. The ratio of FBG/FINS was significant higher in PF-EO rats than in PF-SF rats. Ovarian morphology showed that the numbers of preantral follicles and atretic follicles decreased significantly, and the numbers of antral follicles and corpora lutea increased significantly in the rats of PF-EF and PF-EO.

**Conclusion:**

By combination of high-fat diet and testosterone injection, the obese PCOS rat model is conformable with the lifestyle habits of fatty foods and insufficient exercise, and has metabolic and reproductive characteristics of human PCOS. This model can be applied to study exercise intervention.

## Introduction

Polycystic ovary syndrome (PCOS) is a heterogeneous syndrome that is characterized by anovulation, clinical and/or biochemical signs of hyperandrogenism, and abnormal ovarian morphology [1]. It is the most common endocrine disorder in women of reproductive age, affecting 5–10% of women in this age group [2].

Various methods have been used to induce PCOS in rodents, including exposure to androgens, estrogens, aromatase inhibitors, antiprogesterone agents, constant light and genetic modifications [3]. Estradiol valerateis often used to make a PCOS rat model, which has several characteristics of acyclicity and polycystic ovarian changes without the typical metabolic disturbances of human PCOS [4]–[7]. Letrozole, a nonsteroidal aromatase inhibitor, blocks the conversion of testosterone to estradiol and also induces PCOS in 6-week-old female rats. Continuous administration of letrozole, 200 mg/d, to female rats for 90day starting before puberty results in a PCOS model with reproductive and metabolic features of the syndrome [8]. The letrozole model targets the study of aromotase deficiency–induced classic PCOS [3] and can used for studying the mechanism for the complex pathogenesis of PCOS [8]. Continuous exposure to dihydrotestosterone (DHT) can develop an obese PCOS rat model which has typical hormonal disorders and ovarian morphological changes [9]. The DHT model may be suitable for studies of both ovarian and metabolic features of the syndrome. The insulin sensitivity and estrus cyclicity in DHT induced PCOS rat model can be improved by running wheel exercise [10], although circulating sex hormones were not measured. The model by administration of testosterone to immature female rats seeks to incorporate both the morphological changes as well as the hormonal disorders seen in PCOS. It was demonstrated that testosterone administration blocked the ovulatory process in the model of immature rats. After injection of testosterone propionate for up to 28 days, typical high insulin blood levels and the fasting glucose/insulin ratio was dramatically reduced in the animals. Nevertheless, because a large pool of primordial follicles was still well preserved as well as a small fraction of preantral and antral follicles were still remained, this model can lend itself for the therapy of PCOS phenotype [11]. In our previous study, swimming training for 2 weeks improved significantly body weight index, insulin resistance and the androgen level in PCOS rat model induced by testosterone injection [12].

The pathogenesis of PCOS is poorly understood until now. Starting from early life and extending through lifecycle, environmental insults may affect susceptible women who finally demonstrate the clinical phenotype of PCOS. High-fat diet and sedentary lifestyle emerge as the major environmental determinant of PCOS [13]. Many studies have reported that the rats fed with high-fat diet develop insulin resistance which is one of the important features of PCOS [14]–[16]. Many researchers have demonstrated that the improvement of insulin sensitivity decreased androgen concentration [17]–[19]. Exercise training can significantly improve insulin resistance, thus reducing serum levels of androstenedione and dehydroepiandrosterone [20]. However, several studies have demonstrated that simple dietary intervention did not improve insulin resistance. A European randomized dietary intervention study for 12 weeks had confirmed that overall reduction of dietary SFA had no effect on insulin sensitivity in weight-stable obese subjects [21]. Rajaie et al also found that compared a calorie-restricted moderately restricted carbohydrate and high fat diet (43%–47% of total calories as carbohydrate and 36%–40% as dietary fats) with a calorie-restricted high-carbohydrate and low fat diet (60%–65% of energy from carbohydrates and 20%–25% from fats), there was not statistically different changes in fasting plasma glucose and insulin [22]. Therefore, in the lifestyle interventions, exercise is used as an important measure and diet is considered as a supporting factor. According to unhealthy lifestyle such as high-fat diet and lack of exercise existing in human PCOS, the purpose of the present study is to develop new animal model to fit exercise intervention. In this present study, obese PCOS rat model manifesting hyperinsulinemia, hyperandrogenemia and morphological feature of polycystic ovaries was successfully created by the combination of high-fat diet and testosterone propionate injection, and then intervened by exercise training with diet structure adjustment. The results indicated that exercise intervention can effectively improve the reproductive endocrine environment of the obese PCOS model rats.

## Materials and Methods

### Ethics Statement

Thirty-six Wistar female rats (50–70 g, 21 days old) were purchased from Shanghai SLC Co. (Shanghai, China), kept in temperature-controlled rooms (22°C), with light from 08:00 to 20:00 h and dark from 20:00 to 08:00 h and free access to water. The experimental protocols were approved and strictly followed by the Institutional Animal Care and Use Committees of Nanjing Medical University (permit number: NJMU/IACUC_20120210_01) in strict accordance with the Guidelines for the Care and Use of Laboratory Animals (National Research Council of People's Republic of China, 2010).

### Study procedure

**Figure 1:**
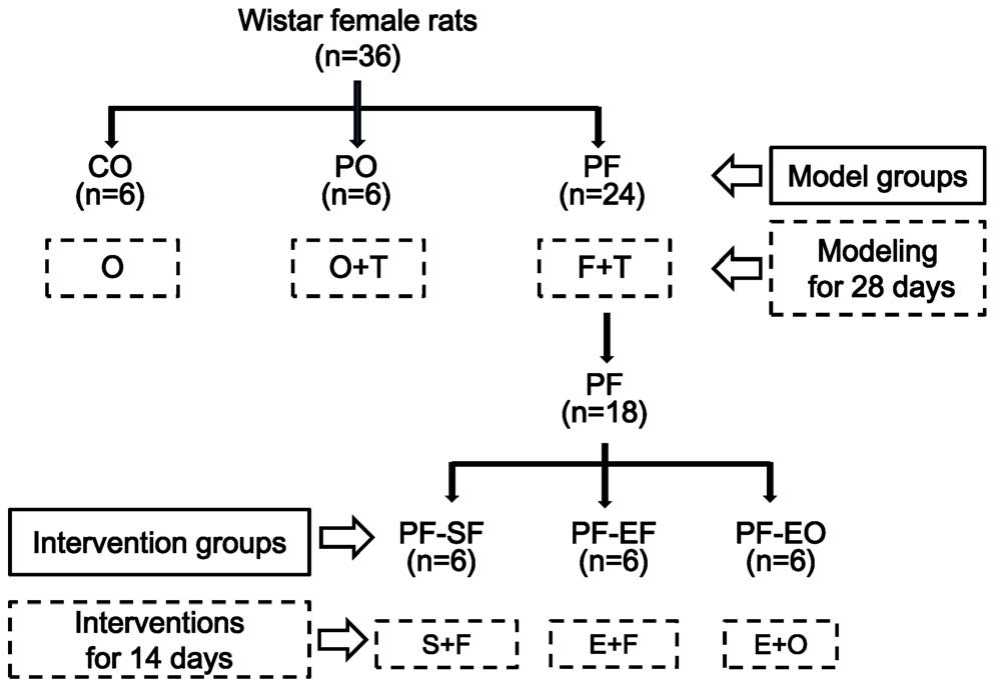
The experiment protocol. O: ordinary diet; F: high fat diet; T: testosterone propionate; CO: control group with ordinary diet; PO: PCOS model with ordinary diet; PF: PCOS model with high-fat diet; S: no exercise; E: exercise; PF-SF: sedentary with high-fat diet in PF rats; PF-EF: exercise with high-fat diet in PF rats; PF-EO: exercise with ordinary diet in PF rats.

All rats were randomly divided into three experimental groups (Fig. 1): PCOS model with high-fat diet (PF, n = 24), PCOS model with ordinary diet (PO, n = 6), and control group with ordinary diet (CO, n = 6). PF rats were fed with high-fat diet for 28 days. CO rats were fed with ordinary diet. High-fat diet composed of 22% fat, 48% carbohydrate, and 20%protein with total calorific value 44.3KJ/Kg [23] and ordinary diet composed of 5% fat, 53% carbohydrate, 23% protein, with total calorific value 25KJ/Kg were ordered in Shanghai SLC Co. (Shanghai, China). At the same time, these rats in PF and PO were subcutaneously injected daily at 8:00 for 28 days with testosterone propionate of 1 mg/100 g body weight dissolved in tea oil [11]. The rats in CO were injected daily with the same volume of tea oil alone. At the day after the end of 28 days of injection, total 18 rats from PF, PO and CO with 6 rats in each group were sacrificed by cervical dislocation.

Then, the remaining 18 rats in PF were randomly assigned to three groups (Fig. 1): exercise with high-fat diet (PF-EF, n = 6), exercise with ordinary diet (PF-EO, n = 6), sedentary with high-fat diet (PF-SF, n = 6). The rats in PF-EF and PF-SF continued feeding with high-fat diet, and the rats in PF-EO were fed with ordinary diet for two weeks. The exercise rats in PF-EF and PF-EO were forced to swim simultaneously without a load at with 120 min/d and 6 d/week for two weeks in a barrel filled with water maintained at 33–35°C to a depth of 40–50 cm, in which the area for every rat is enough to swim freely. The non-exercise rats in PF-FS were placed in shallow water at 33–35°C, 120 min, 6 day/week for two weeks of housing [12].All the rats were sacrificed by cervical dislocation between 08:00 and 11:00 AM under fasting condition at24 h after the end of the last training session with 3% pentobarbital abdominal anesthesia. Blood and ovary samples were collected from all animals.

### Measurements of body weight, body length and ovary weight

Body weight and length of rats were measured weekly from 21days of age. Body length was defined as the distance from nose to anus of rats. Lee index reflects the body fat as a parameter [LI =  body weight (g) ^1/3^×1000/body length (cm)] [11]. Bilateral ovaries of one rat were weighed, in which the mean values were regarded as the ovary weight. Ovarian organ index  =  ovary weight/body weight ×10^−3^ [12].

### Vaginal smears

Vaginal smears were monitored daily to determine the stage of cyclicity from 42 days of age to the end of experiment. In this procedure, smears obtained by vaginal washing were dyed with methylthioninium chloride and then analyzed under a microscope for the predominant cell type in vaginal smears. The estrus cycle experiences four stages of proestrus, estrus, metoestrus, and diestrus. Cycles with duration of 4–5 days were considered as regular [3]. At the beginning of experiment, all rats showed regular cycles.

### Oral glucose tolerance test (OGTT)

Before sacrificed, all rats were fasted overnight and infused intragastrically with 2 g glucose per kilogram of body weight. Blood samples were collected at 0 min, 60 min and 120 min after oral glucose used to evaluate fasting blood glucose (FBG), 1 h postprandial blood glucose (PBG_1_) and 2 h postprandial blood glucose (PBG_2_), respectively [23].

### Serum analysis

Blood samples obtained from all rats fasting for 12 h were centrifuged at 2500 *g* for 10 min and stored at −80°C. Fasting serum levels for insulin (FINS), estrogen (E_2_), progesterone (P), and testosterone (T) were determined using correspondent commercial RIA kits (Northern biological technical institute, Beijing, P. R. China). Fasting blood glucose (FBG) was analyzed by GOD-PAP. Insulin resistance index is defined as the ratio of FBG/FINS [11].

### Ovarian morphology analysis

When rats were sacrificed, the ovaries were dissected from connective tissue, fixed in 10% formalin for 24 h, dehydrated in increasing concentrations of ethanol, followed by immersion in xylene, and embedded in paraffin. The ovaries were longitudinally and serially sectioned at 4 µm from the center. Serial sections were mounted on 3 slides, deparaffinized with xylene, hydrated with successive decreasing concentrations of ethanolin water, and then stained with hematoxylin and eosin (HE). The section with the largest area was chosen for analysis to count the numbers of preantral, antral and atretic follicles, and corporalutea [24]. Atretic follicles were confirmed when one of the following characteristics was observed, such as the presence of pyknosis in the granulose cell, granulosa cells present in the follicular fluid, or theca cell hypertrophy. Sections were photographed with a camera (Olympus BX51, Japan) at ×40, ×100, and ×200 magnifications. The area of the ovary was determined with a calibrated scale tool in the virtual microscope. Then, the areas of the largest section (at ×40 magnification), all corporalutea (at ×100 magnification), and thickness of theca cell layer and granulosa cell layer (from 3 visual fields of follicles at ×200 magnification) were measured with Image-Pro plus 6.0.

### Statistical analyses

Data were expressed as means ±SD. Statistical evaluations were performed with SPSS software (version 16.0, SPSS, Chicago, IL, USA). All the data were analyzed by one-way ANOVA, and p<0.05 was considered significant. When the ANOVA revealed significant differences among three experimental groups, post hoc analysis was performed by using Bonferroni's method of multiple comparisons (3 repeated tests; p<0.0167 was considered significant for each test).

## Results

### Vaginal smears

**Figure 2:**
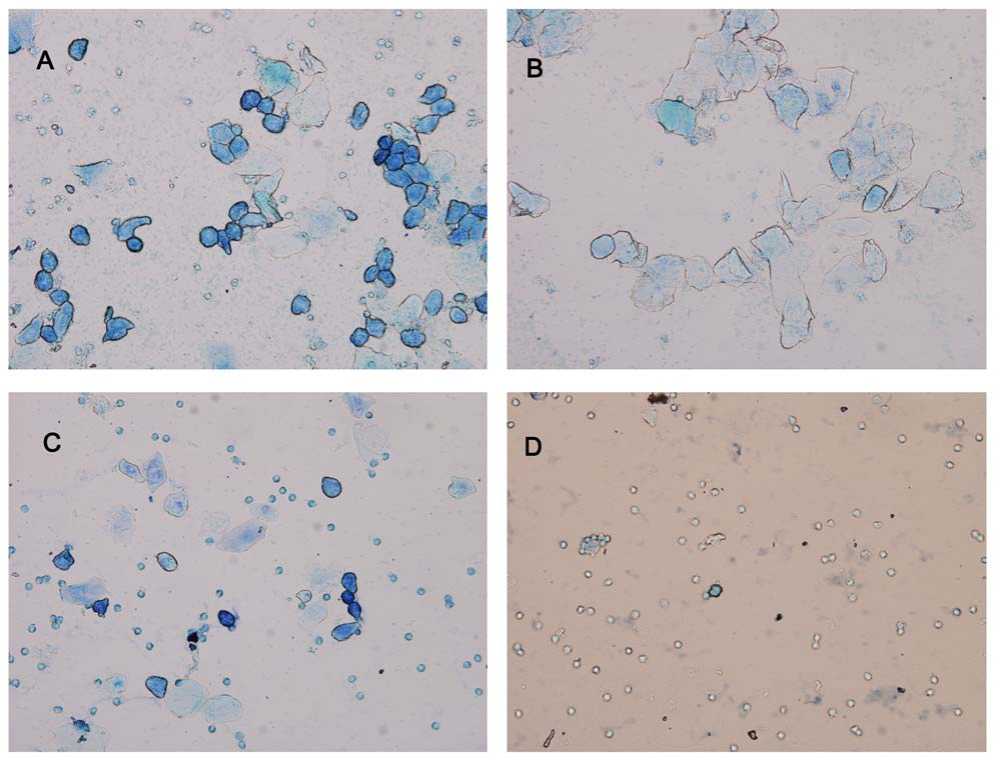
Figures and cell types of vaginal smears. **A**: preoestrus, loaded mainly with epithelial cell; **B**: estrus, loaded mainly with keratinocytes; **C**:metoestrus,displaying epithelial cell, keratinocytes, and leukocytes; **D**:diestrus, displaying full of leukocytes (magnification ×100).

The vaginal smears were monitored during whole experiment. The vaginas in all rats mostly opened at 40 days old. The regular estrous cycle of 4–5 days was observed in control (Fig. 2). However, there were many white cells, less epithelial cell, and keratinocytein vaginal smears of PO and PF rats, indicating these rats persisted in diestrus and no ovulation happened. After 2 weeks, the rats in PF-EF and PF-EO restored to normal estrous cycles, while the rats in PF-SF still showed persistent diestrus.

### Changes of metabolic and endocrine variables in PCOS model induced by high-fat diet and testosterone propionate

The metabolic and endocrine variables were measured on the 28^th^ day of experiment in three groups of CO, PO and PF. Body weight and Lee index in PF significantly increased in comparison with CO and PO. The levels of FBG were significantly lower in PF than in CO. The levels of FINS were significantly higher in PF than in PO and CO, while ratios of FBG/FINS in PF significantly decreased in comparison with other two groups. Serum levels of E_2_ and T were significantly higher in PF and PO than in CO (Table 1).

**Table 1:** Changes of metabolic and endocrine variables in obese PCOS model induced by high-fat diet.

	CO	PO	PF
Body weight(g)	138.33±10.51	149.83±10.07	172.33±11.52 * #
Body length(cm)	17.22±0.64	17.96±0.64	17.65±0.75
Lee Index	300.35±3.61	295.76±3.90	315.36±8.39 * #
FBG (mmol/l)	5.38±0.55	4.88±0.46	4.38±0.60 *
PBG_1_ (mmol/l)	9.15±0.53	8.72±1.35	9.43±0.82
PBG_2_ (mmol/l)	6.95±0.61	6.43±0.71	7.53±1.56
FINS (uIU/ml)	21.63±4.02	23.90±4.03	50.58±6.86* #
FBG/FINS	0.26±0.05	0.23±0.09	0.10±0.03 * #
E_2_ (ng/ml)	1.60±0.31	2.41±0.56 *	2.61±0.28 *
P (ng/ml)	0.39±0.22	0.31±0.16	0.25±0.06
T (ng/ml)	0.17±0.08	28.32±7.24 *	31.92±6.31 *

Values are means ± SD.

*p<0.0167 versus CO by Bonferoni post-hoc test.

# p<0.0167 versus PO by Bonferoni post-hoc test.

### Changes of ovarian morphological parameters in PCOS model induced by high-fat diet and testosterone propionate

**Figure 3:**
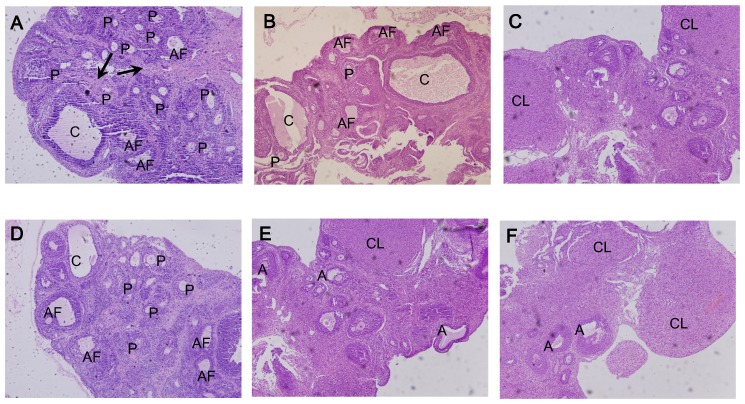
Ovarian morphology of rats from three groups (×100). **A**: **PF**. A lot of preantral follicles (P) and atretic follicles (AF) with enlargement of stroma (arrow) and cystic follicles (C) are shown in the section. **B**: **PO**. A lot of preantral follicles (P), atretic follicles (AF) and cystic follicles(C) are shown in the section. **C**: **CO**. Various stages of development follicles and some corpora lutea (CL) are shown. **D**: **PF-SF**. A lot of preantral follicles (P) and atretic follicles (AF), and cystic follicles(C) are shown in the section. **E**: **PF-EF**. A lot of antral follicles (A) and a bit of corpora lutea (CL) are shown in the section. **F**: **PF-EO**. A lot of antral follicles (A) and a bit of corpora lutea (CL) are shown in the section.

Ovarian morphological parameters were measured on the 28^th^ day of experiment in three groups of CO, PO and PF (Fig. 3). Ovary organ index and ovarian areas were significant lower in PF than in CO. Corpora lutea in PF and PO were not observed. In comparison with CO group, thickness of theca layer, numbers of preantral follicle, numbers of atretic follicle, total numbers of follicle and ratios of numbers of atretic follicle to total numbers of follicles significantly increased in PF and PO, while thickness of granular layer significantly decreased in PF and PO (Table 2).

**Table 2:** Changes of ovarian morphological parameters in PF.

	CO	PO	PF
Ovary weight(mg)	18.55±3.41	16.09±2.37	15.80±1.79
Ovarian organ index(×10^−3^)	0.14±0.03	0.12±0.02	0.09±0.02 *
Ovarian area(mm^2^)	2.79±0.49	1.77±0.88	1.07±0.18 *
Corpora lutea area(mm^2^)	1.04±0.48	0.00±0.00 *	0.00±0.00 *
Thickness of granular layer(µm)	35.05±4.63	24.36±5.14 *	24.03±4.91 *
Thickness of theca layer(µm)	14.56±1.28	19.04±2.39 *	19.63±1.94 *
Numbers of preantral follicle	3.67±1.63	7.67±1.86 *	10.20±1.30*
Numbers of antral follicle	2.83±2.40	2.83±1.47	1.80±0.84
Numbers of atretic follicle	1.17±1.17	7.00±1.90*	9.00±3.67 *
Total numbers of follicle	7.67±3.67	17.50±3.94 *	21.00±2.65 *
Numbers of corpora lutea	4.00±1.26	0.00±0.00 *	0.00±0.00 *
Ratio of corpora lutea area to ovarian area	0.37±0.16	0.00±0.00 *	0.00±0.00*
Ratio of numbers of corpora lutea to total numbers of follicle	0.62±0.34	0.00±0.00 *	0.00±0.00 *
Ratio of numbers of atretic follicle to total numbers of follicle	0.13±0.12	0.40±0.08 *	0.42±0.12*

Values are means ± SD.

*p<0.0167 versus CO by Bonferoni post-hoc test.

### Effects of exercise and diet on metabolic and endocrine variables in PF rats

The metabolic and endocrine variables were measured at the end of experiment in PF-SF, PF-EF and PF-EO. Body weight was significantly lower in PF-EF and PF-EO than in PF-SF. The levels of FBG were significant higher in PF-EO than in PF-SF. The levels of PBG_1_, PBG_2_ and FINS were significantly lower in PF-EF and PF-EO than in PF-SF, while the levels of PBG_2_ were significantly lower in PF-EO than in PF-EF. In comparison with PF-SF, the ratio of FBG/FINS increased significantly in PF-EO. Serum levels of T were significantly lower in PF-EF and PF-EO than in PF-SF. There were no statistical differences in serum levels of E_2_ and P in the three groups of PF-SF, PF-EF and PF-EO (Table 3).

**Table 3:** Effects of exercise and diet on metabolic and endocrine variables in PF rats.

	PF-SF	PF-EF	PF-EO
Body weight(g)	213.00±9.86	188.50±7.77*	174.33±11.81*
Body length(cm)	19.31±0.61	19.24±0.49	18.69±0.57
Lee Index	309.21±5.85	298.03±8.95	299.01±9.08
FBG(mmol/l)	4.00±0.32	4.20±0.42	4.75±0.31*
PBG_1_ (mmol/l)	9.11±0.33	7.70±0.49*	7.01±0.64*
PBG_2_ (mmol/l)	9.20±1.42	7.40±0.94*	5.67±0.43* #
FINS(uIU/ml)	21.75±0.73	17.63±1.66*	16.89±1.20*
FBG/FINS	0.18±0.02	0.24±0.04	0.29±0.05*
E_2_ (ng/ml)	1.72±0.31	1.36±0.51	1.30±0.28
P (ng/ml)	0.44±0.10	0.54±0.08	0.59±0.18
T (ng/ml)	0.18±0.02	0.08±0.04 *	0.07±0.03*

Values are means ± SD.

*p<0.0167 versus PF-SF by Bonferoni post-hoc test.

# p<0.0167 versus PF-EF by Bonferoni post-hoc test.

### Effects of exercise and diet on ovarian morphologic parameters in PF rats

Ovarian morphological parameters were measured on the end of experiment in PF-SF, PF-EF and PF-EO. In comparison with PF-SF, ovary organ index, ovarian areas, numbers of antral follicle, and numbers of corpora lutea significantly increased, while thickness of theca layer, numbers of preantral follicle, numbers of atretic follicle, total numbers of follicle, and ratio of numbers of atretic follicle to total numbers of follicle significantly decreased in PF-EF and PF-EO. The ratios of numbers of corpora lutea to total numbers of follicles were significantly higher in PF-EF and PF-EO than in PF-SF, and were significantly lower in PF-EF than in PF-EO. Other parameters in corpora lutea areas and thickness of theca layer were significantly higher in PF-EO than in PF-SF (Table 4).

**Table 4:** Effects of exercise and diet on ovarian morphological parameters in obese PCOS rats.

	PF-SF	PF-EF	PF-EO
Ovarian weight(mg)	13.92±1.06	16.02±1.62	16.11±1.57
Ovarian organ index(×10^−3^)	0.07±0.00	0.09±0.01*	0.09±0.01*
Ovarian area(mm^2^)	4.74±0.65	5.73±0.45*	6.17±0.47*
Corpora lutea area(mm^2^)	1.18±0.43	1.52±0.52	2.03±0.42*
Thickness of granular layer(µm)	22.59±3.53	26.04±5.03	29.28±3.35*
Thickness of theca layer(µm)	16.76±1.78	13.96±1.61*	13.37±1.53*
Numbers of preantral follicle	9.83±2.79	5.67±1.75*	3.67±1.21*
Numbers of antral follicle	2.33±1.37	6.17±1.47*	7.17±1.17*
Numbers of atretic follicle	5.33±2.16	2.00±0.89*	1.33±1.03*
Total numbers of follicle	17.50±2.07	13.83±2.23*	12.16±1.47*
Numbers of corpora lutea	3.00±1.10	5.67±0.82*	8.33±2.58*
Ratio of corpora lutea area to ovarian area	0.25±0.11	0.26±0.08	0.33±0.08
Ratio of numbers of corpora lutea to total numbers of follicle	0.17±0.06	0.42±0.08*	0.68±0.17* #
Ratio of numbers of atretic follicle to total numbers of follicle	0.31±0.13	0.14±0.05*	0.11±0.09*

Values are means ± SD.

*p<0.0167 versus PF-SF by Bonferoni post-hoc test.

# p<0.0167 versus PF-EF by Bonferoni post-hoc test.

### Microscopic observation of ovarian section

Follicles in various stages of development and atretic follicle in small amounts were observed on sections from PF-EF, PF-EO and CO. A large number of preantral and atretic follicles and some cystic follicles were observed in PO, PF and PF-SF. The pathological characteristics such as thickened ovarian adventitial coat, obvious demixing between theca interna and theca externa, inspissated theca cell layer, and apparent proliferation of ovarian stroma were observed in PO, PF and PF-SF (Fig. 3).

## Discussion

In this study, the obese PCOS rats were successfully induced by using high-fat diet and testosterone injection at the same time. The PF rats had endocrine and reproductive characteristics such as hyperandrogenemia, hyperinsulinemia, anovulation, and enlarged ovaries with follicular atresia and multiple cysts in the periphery, which is consistent with human PCOS [25]. A difference of approximately 24% in body weight was observed between PF rats and CO rats. This body weight difference has been assumed in the literature as inferring obesity [26]. Except for body weight, the Lee Index in PF rats displayed a statistically significant difference compared with rats of CO and PO. Therefore, PF rats were considered as an obese PCOS model.

Diet, as the major environmental determinant, plays an important role during early stages of human development from prenatal life to puberty[13].The dietary intake of advanced glycated end products(AGEs) such as lipid and protein-rich foods is increasingly recognized as a determinant of metabolic and cardiovascular morbidity [27], [28]. Diamanti-Kandarakis et al. found that the rats with high-fat feeding manifested concurrent increase of testosterone, suggesting an impact of dietary AGEs on ovarian tissue [29]. Androgen excess plays an important role in the pathophysiology of PCOS and has been hypothesized to be the final common pathway for the development of the signs and symptoms of this disorder. The majority (80%) of adults with PCOS have hyperandrogenemia [30]. Long-term administration of androgens can induce visceral obesity and consecutive insulin resistance. Abdominal adiposity, insulin resistance and compensatory hyperinsulinemia could trigger androgenization in women [31]. The predominate increase of TP and E_2_ in PO and PF was considered to be mostly due to persistent TP injection in this present study. Frankset al. reported that the characteristic feature of polycystic ovaries is an apparent failure to select a dominant follicle and the accumulation of antral follicles 2–8 mm in size. It was assumed that this appearance reflects an androgen-induced arrest in antral follicle development [32]. Several studies on rats have demonstrated that continuous exposure of post pubertal animals to steroid hormones, either via regular s.c. dosing or continuous release pellet implant, produces an anovulatory phenotype in these animals [33]. In this present study, ovarian morphological parameters in both PF and PO rats manifested large, atretic antral follicles, follicular cysts with a thickened theca interna cell layer, a diminished granulosa cell compartment, and few fresh corpora lutea, consistent with the research by Beloosesky et al [11]. Ovarian organ index and ovarian area were significantly less in PF rats than in CO rats. These results suggest that a high-fat diet is more likely to form typical PCOS morphological changes in comparison with ordinary diet.

Sedentary behavior, as assessed by sitting time, is increased in human PCOS and is being increasingly recognized as a contributor both to elevated adiposity and to obesity-associated [34]. In this present study, the obese PCOS rat model induced by high-fat diet and testosterone injection imitated successfully the unhealthy lifestyle habits such as high lipid diet and insufficient exercise of human PCOS. Testosterone administration induces insulin resistance, attributed to effects on glucose transport. Another finding was reduced capillary density in the muscle despite increased muscle weight [9]. Previous studies showed that high fat diet of obese mice and humans could lead to increased FFA influx through the portal vein into the liver, which could result in a state of IR [14]. These PF rats manifested significant metabolic characteristics of insulin resistance (increase of FINS and decrease of FBG/FINS) and overweight or obesity (increase of Lee Index) in comparison with PO rats. Insulin resistance has been recognized to contribute not only to metabolic but also to reproductive aspects of the syndrome [13], [35]. Numerous studies also have shown that a high-fat diet can cause insulin resistance in muscles at rest [15]. Although insulin resistance is not enough to become the diagnosis of PCOS, it is present in up to 50% women with PCOS [36]. Overweight or obesity is a common feature of PCOS women [37], [38]. The link between PCOS and overweight or obesity also has been confirmed in adolescent PCOS patients [39].

Exercise based lifestyle modifications are successfully employed to treat obese and overweight women with PCOS [2]. Morifuji et al. reported that intake of dietary soya protein and exercise training by swimming for 2 weeks had an additive effect on decreases of insulin resistant and body fat in rats [40]. Our previous study has shown that exercise for 2 weeks increased insulin sensitivity, decreased serum androgen levels, and recovered normal ovarian morphology in PCOS rats without high-fat diet [12]. In this present study, swimming training for 2 weeks, with or without a high-fat diet, significantly decreased PBG_1_, PBG_2_, FINS and the lever of testosterone, and improved ovarian morphology indexes in PF rats. Lungeret et al. suggested the use of FINS as a simple screening test for assessment of insulin resistance in women with PCOS [36]. In this present study, the decrease of FINS after swimming training indicated the improvement of insulin resistance to be associated with exercise rather than diet.

Overweight and obesity impact the clinical reproductive and metabolic features of the syndrome. Long-term complications of obesity such as cardiovascular risk and diabetes, as well as those over the short term in reproductive function, are significantly improved by lifestyle modification. Weight loss decreases significantly hyperinsulinemia and hyperandrogenism in women with PCOS [42]. High-fiber diet and low-fat diets enriched with monounsaturated fats may help to reduce body weight [43]. Several studies have paid close attention to impacting of low carbohydrate and standard dietary intervention regimens on both amount of weight lost as well as specific metabolic and endocrine features of human PCOS [44]. In a short-term crossover trial of human PCOS within 16 days, fasting insulin was lower following a low carbohydrate (43%) diet relative to a standard carbohydrate diet (56% carbohydrate, 31% fat, 16% protein). Fasting glucose, insulin sensitivity, and the circulating concentrations of reproductive hormones were not significantly affected by the intervention [45]. In our present study, the ratio of fasting glucose to fasting insulin in PF-EO rats was significantly higher than that in PF-SF rats, and the levels of PBG2 were significantly lower in PF-EO rats than in PF-EF rats. These results suggested that dietary constitutes had a certain influence on blood glucose.

The appearance of corpora lutea is considered as occurrence of ovulation. In this present study, only a few corpora lutea were observed in PF-SF. Four rats in PF-SF showed persistent diestrus and the other two PF-SF rats showed irregular cycles. Manneras et al. also reported that the PCOS rat model induced by DHT had irregular cycles [9]. Their ovaries had large, atretic antral follicles, follicular cysts with a thickened theca interna cell layer, and few fresh corpora lutea, similar to our present results. In this present study, swimming training for 2 weeks, with or without high-fat diet, increased significantly the numbers of corpora lutea, the ratio of numbers of corpora lutea to total numbers of follicle and numbers of antral follicle, and decreased significantly numbers of preantral follicle and atretic follicle, according with Manni's research [7]. These results suggest that exercise is conducive to promoting follicular maturation and ovulation. The possible explanation for the improvement of cyclicity is that decreased sympathetic activity will have a direct impact on the ovaries and affect the sex steroid synthesis pathways. It has been shown that physical exercise can decrease sympathetic nerve activity, improve menstrual frequency, and decrease the levels of several sex steroids in women with PCOS [10]. In addition, significant increases in thickness of granular layer and corpora lutea area were found in PF-EO rats but not in PF-EF rats. The ratio of numbers of corpora lutea to total numbers of follicle was also significantly higher in PF-EO rats than in PF-EF rats, suggesting that dietary adjustments had an additional role on promotion of ovulation. Lifestyle intervention of human PCOS has been widely accepted. From our research results, exercise training may play a more important role on reproductive/endocrine outcomes.

Different mechanisms of reproductive function improved by exercise may be involved to explain their beneficial effects on glucose/insulin homeostasis. Insulin sensitivity improvement itself is the pivotal factor involved in the restoration of ovarian function. Several reports have demonstrated that loss of body weight is related to increase of insulin sensitivity [46], [47]. Exercise could ameliorate insulin sensitivity not only by a large reduction of body weight and visceral fat [48] but also by cellular muscle metabolism enhancement. Skeletal muscle is the main site of glucose deposition implicated in insulin resistance, and it is well known that physical exercise influences the expression and/or activity of proteins involved in insulin signal transduction in skeletal muscle [49] such as the translocation of GLUT-4 glucose transporters to the plasma membrane and transverse tubules [41]. Previous studies demonstrated improvement of insulin resistant induced by exercise was accompanied with the concurrent decrease of hyperandrogenism [12], [20]. It is speculated that androgen may be involved in some sections of glucose metabolism, which needs to be confirmed by further studies.

Conclusively, our present study demonstrated that obese PCOS rat model created successfully by a combination of high-fat diet and testosterone injection was conformable with metabolic and reproductive characteristics of human PCOS and can be used for mechanism study of exercise intervention on reproductive/endocrine outcomes of PCOS.
